# Genome-wide association study identifies CD1A associated with rate of increase in plasma neurofilament light in non-demented elders

**DOI:** 10.18632/aging.102066

**Published:** 2019-07-11

**Authors:** Zuo-Teng Wang, Shi-Dong Chen, Wei Xu, Ke-Liang Chen, Hui-Fu Wang, Chen-Chen Tan, Mei Cui, Qiang Dong, Lan Tan, Jin-Tai Yu, Alzheimer’s Disease Neuroimaging Initiative

**Affiliations:** 1 Department of Neurology, Qingdao Municipal Hospital, College of Medicine and Pharmaceutics, Ocean University of China, Qingdao, China; 2 Department of Neurology and Institute of Neurology, Huashan Hospital, Shanghai Medical College, Fudan University, Shanghai, China; 3 Department of Neurology, Qingdao Municipal Hospital, Qingdao University, Qingdao, China

**Keywords:** genome-wide association study, plasma NFL, non-demented elders, CD1A, genetic factors

## Abstract

As a marker of neuroaxonal injury, neurofilament light (NFL) in blood is robustly elevated in many neurodegenerative conditions. We aimed to discover single nucleotide polymorphisms (SNPs) associated with longitudinal changes in plasma NFL levels that affect the risk of developing neurodegenerative disease and clinical disease progression. 545 eligible non-Hispanic white participants from the Alzheimer’s Disease Neuroimaging Initiative (ADNI) with longitudinal plasma NFL data were included. Three SNPs (rs16840041, p=4.50×10^-8^; rs2269714, p=4.50×10^-8^; rs2269715, p=4.83×10^-8^) in *CD1A* were in high linkage disequilibrium (LD) and significantly associated with the increase in plasma NFL levels. We demonstrate a promoting effect of rs16840041-A on clinical disease progression (p = 0.006). Moreover, the minor allele (A) of rs16840041 was significantly associated with accelerated decline in [^18^F] Fluorodeoxyglucose (FDG) (estimate -1.6% per year [95% CI -0.6 to -2.6], p=0.0024). *CD1A* is a gene involved in longitudinal changes in plasma NFL levels and AD-related phenotypes among non-demented elders. Given the potential effects of these variants, *CD1A* should be further investigated as a gene of interest in neurodegenerative diseases and as a potential target for monitoring disease trajectories and treating disease.

## INTRODUCTION

Neurofilament light (NFL) is gaining increasing attention as a potential biomarker of neuroaxonal injury which is the pathological substrate for permanent disability in various neurodegenerative diseases. Regardless of clinical diagnosis, high levels of NFL are general indicators of axonal damage. Accumulating evidence has indicated that the plasma NFL is useful for predicting and monitoring progression in various neurodegenerative diseases, including Alzheimer’s disease (AD) [1]. Increase in plasma NFL is well established in neurodegenerative pathology, but the genetic contribution to this change needs further research [2–4]. Previous studies suggested that the candidate biomarkers can be used as endophenotypes in genome-wide association study (GWAS) [5–7]. In a previous study, baseline plasma NFL data were used to explore genetic factors [8]. Since inter-individual variability may exist in the disease trajectories, cross-sectional data have limitations with respect to the evaluation of clinical disease progression. Longitudinal changes in plasma NFL may provide important insights into genetic mechanism underlying these diseases. All samples from the subjects in Alzheimer’s Disease Neuroimaging Initiative (ADNI) cohort were measured longitudinally for changes in plasma NFL levels. Thus, we can use the plasma NFL to carry out longitudinal tracking of AD-related indicators over extended periods of time. In this study, we present the first GWAS of the rate of change in plasma NFL among non-demented elders (cognitive normals (CN) or those diagnosed with mild cognitive impairment (MCI)). We hope to identify novel variants specific to the longitudinal changes in plasma NFL levels.

## RESULTS

### Characteristics of included subjects

After quality control (QC), 545 non-Hispanic white participants from the ADNI with longitudinal plasma NFL data were included. Detailed information of included subjects is presented in Table 1. All available longitudinal plasma NFL data were included in the linear mixed effects models (adjusted for age at baseline, diagnosis, and marital status). We obtained a residual plasma NFL change rate for each individual. Continuous quantitative change rates were primary outcome measures used in the genetic association studies.

**Table 1:** Demographic information of the studies subjects.

**Baseline diagnosis**	**HC**	**MCI**	**Total**
n	224	321	545
Age at baseline (years), mean ± SD	74.81±5.36	71.69±7.35	72.85±6.78
Gender, male/female	118/106	189/132	307/238
*APOE4* status (0/1/2)	80/24/2	172/118/31	340/169/36
Follow-up years, mean ± SD	4.85±0.57	3.75±1.81	4.20±1.10
Mean annual changes in plasma NFL levels (pg/ml·year), mean ± SD	−0.11±1.56	−0.22±1.51	−0.17±1.51

Abbreviations: AD, Alzheimer’s disease; APOE, Apolipoprotein E; HC, healthy control; MCI, early mild cognitive impairment; NFL, neurofilament light; SD, standard deviationa.

### Single nucleotide polymorphisms (SNPs) associated with the rate of increase in plasma NFL

**Figure 1:**
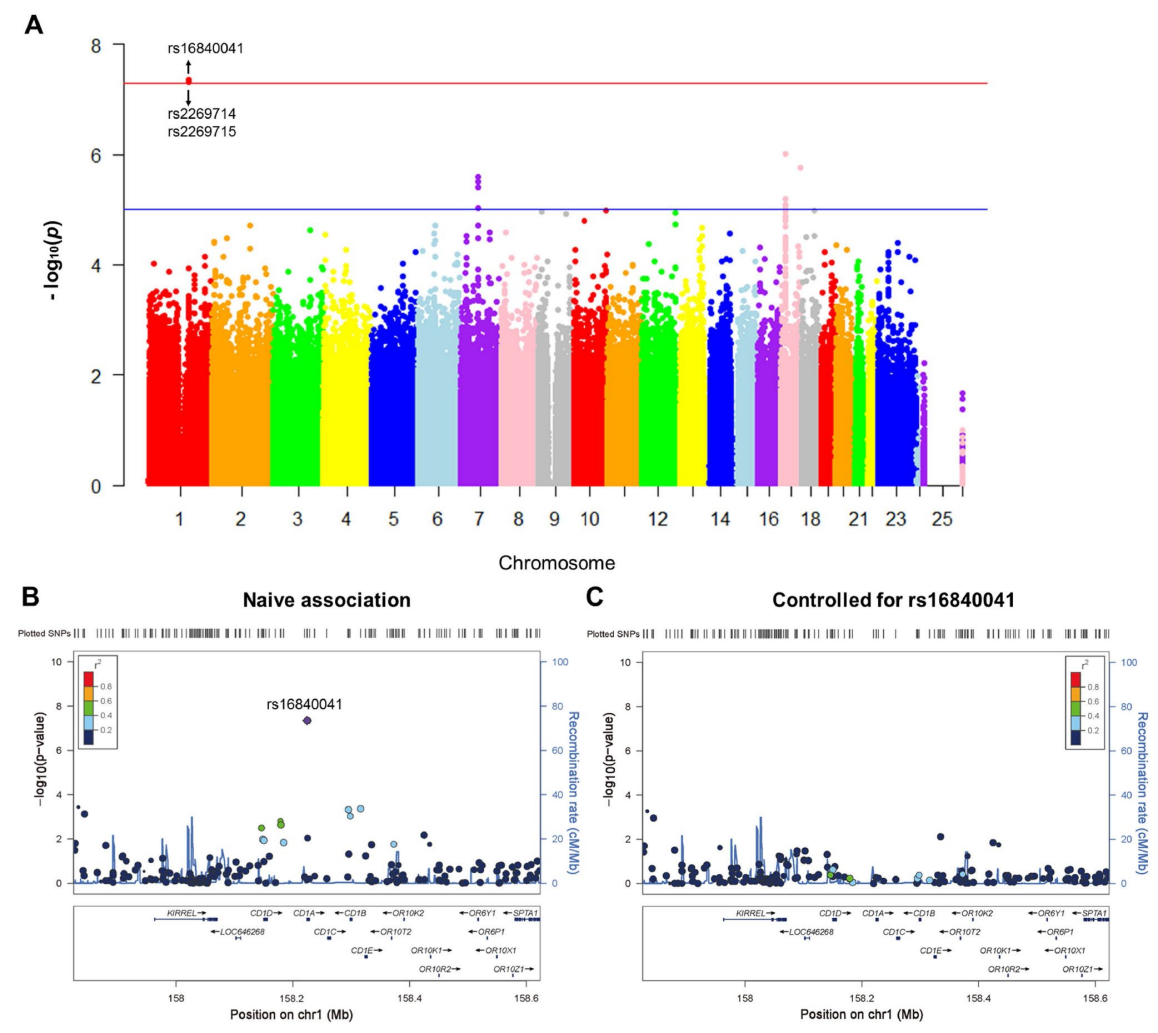
Genome-wide signal intensity (Manhattan) plots showing the -log_10_ (p value) for individual single nucleotide polymorphisms (**A**). Regional association results for the 158 Mb to 158.6 Mb region of chromosome 1 (**B**). Association results for 158 Mb to 158.6 Mb region of chromosome 1 controlling for rs16840041 (**C**).

A total of 1,231,747 genotyped variants were included in GWAS. Three SNPs (rs16840041, p=4.50×10^-8^; rs2269714, p=4.50×10^-8^; rs2269715, p=4.83×10^-8^) were significantly associated with the rate of increase in plasma NFL (Figure 1A and Table 2). Other SNPs with suggestive associations are listed in Supplementary Table 1. Quantile-quantile (Q-Q) plot shows no evidence of population stratification, as most of the observed p-values do not deviate from the expected line (Supplementary Figure 3). The Haploview software was used to conduct linkage disequilibrium (LD) analysis between these SNPs. Rs16840041 was in high LD (r^2^>0.8) with other two SNPs (rs2269714 and rs2269715) in *CD1A* (Supplementary Figure 4).

**Table 2:** Top SNPs associated with the rate of change in plasma NFL.

**SNP**	**CHR**	**Gene**	**Observed MAF**	**SNP Type/Location**	**Beta**	**P values**
rs16840041	1	*CD1A*	0.06	intron	1.042	4.50×10^-8^
rs2269714	1	*CD1A*	0.06	intron	1.042	4.50×10^-8^
rs2269715	1	*CD1A*	0.06	intron	1.040	4.83×10^-8^

Abbreviations: NFL, neurofilament light; CHR, chromosome; MAF, minor allele frequency; SNP, single nucleotide polymorphism.

**Figure 2:**
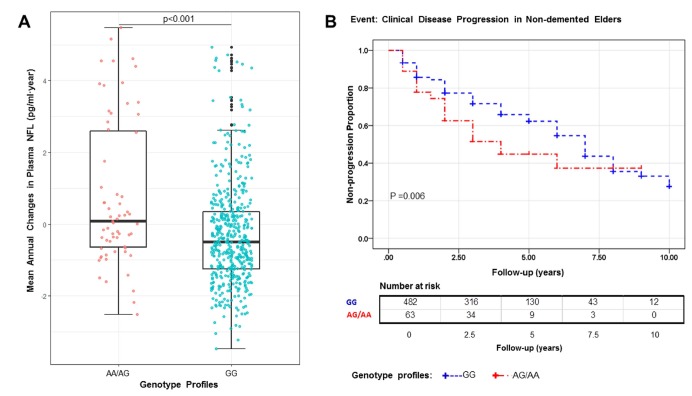
The A allele is associated with a significant increase in plasma NFL (P<0.001), P value in the plot was computed from linear regression model after adjusting for age, diagnosis, and *APOE4* status (**A**). Kaplan–Meier survival curves for the probability of clinical disease progression according to different rs16840041 genotypes. Numbers of individuals at risk at each time interval are shown in the table. Survival time was calculated as the interval from the initial baseline evaluation to the clinical disease progression. AG/AA genotype is associated with an increased risk of clinical disease progression (P = 0.006) (**B**).

In the *CD1A* region, several SNPs in LD with rs16840041 showed values of p < 0.001 for the longitudinal changes in plasma NFL (Figure 1B). However, after controlling for the genotypes of rs16840041, no strong associations remained in this region (Figure 1C), indicating that all the associations in this locus were driven by the three SNPs. Moreover, we identified that the minor allele of rs16840041-A was associated with a significant increase in plasma NFL levels (Figure 2A).

### *CD1A* rs16840041-A affects the risk of clinical disease progression

Figure 2B shows Kaplan–Meier survival curves for the probability of clinical disease progression in the different rs16840041 genotype subgroups. The GG group was significantly associated with longer estimated time of clinical disease progression (6.26 ± 0.19 years, 95% confidence interval (CI) 5.88-6.64), compared with the AA/AG group (4.86 ± 0.50 years, 95% CI 3.90–5.82, p = 0.006). In Cox regression models (adjusted for age, diagnosis and *APOE4* status), the individuals with AA/AG genotype had higher risk of progression to AD (hazard ratio 1.63, 95% CI 1.12-2.36, p = 0.010) (Supplementary Table 2).

### Impact of rs16840041 on other AD-related phenotypes

**Figure 3:**
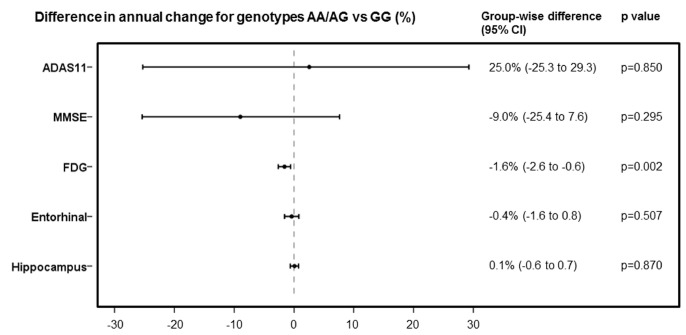
Comparison of rates of change in ADAS11, MMSE, FDG, entorhinal cortex volume and hippocampus volume, expressed as differences in annual percentage changes, with 95% CIs, between AA/AG and GG genotypes.

In the post hoc analyses, we identified 355 subjects in all diagnostic groups with longitudinal [^18^F] Fluorodeoxyglucose (FDG) data available for analysis. Subjects with AA/AG genotype showed significantly faster rates of [^18^F] FDG decline than did those with GG genotype (AA/AG vs GG, estimate -1.6% per year [95% CI -0.6 to 2.6], p=0.0024) (adjusted for baseline age, diagnosis, and *APOE4* status). But for 11-item Alzheimer's Disease Assessment Scale (ADAS11), Mini-Mental State Examination (MMSE), and the volume of AD-related brain regions, we did not observe any significant differences in the rates of change between AA/AG and GG genotypes (Figure 3).

## DISCUSSION

In this study, we identified significant associations between genetic variants and the rates of change in plasma NFL among non-demented elders. Three SNPs (rs16840041, rs2269714 and rs2269715) within *CD1A* in high LD were associated with increased plasma NFL levels. In the other longitudinal frameworks, individuals with the minor alleles showed a higher risk of cognitive decline, and the minor alleles (rs16840041, A; rs2269714, T; rs2269715, G) were significantly associated with an accelerated decline of [^18^F] FDG in the entire cohort.

The three variations are located on chromosome 1q23.1 within *CD1A* region. The *CD1A* gene encodes a member of the CD1 family (CD1A, CD1B, CD1C, CD1D, and CD1E) of transmembrane glycoproteins [9]. CD1A proteins are important molecules presenting glycolipid and lipid antigens of microbial origin or themselves to T cells [10, 11]. Thus, T cells can sense and respond to changes in lipid repertoire (inflammation, infection and malignancies) [12].

Self-reactive T cells (specific for self-glycosphingolipids) were identified in multiple sclerosis (MS) patients. These T cells were restricted by all types of CD1 molecules [13]. CD1A expression was significantly increased in MS patients [14, 15]. Moreover, Caporale et al.’ study suggested that *CD1A* gene polymorphisms are associated with susceptibility to MS [16]. The increased CD1A expression can cause inappropriate presentation of self-lipid antigen and may be one of the pathogenetic mechanisms leading to MS. In addition to self-proteins, self-glycolipids may represent the potential source of autoantigens recognized by T cells in autoimmune diseases [13]. Once activated, CD1self-reactive T cells could regulate both cell-mediated and humoral immune responses [17]. It has been reported that several autoimmune diseases and neurodegenerative diseases (AD, Parkinson disease (PD), and frontotemporal dementia (FTD)) share the same genetic pathways [18–20].

Previous studies have identified that the minor allele (T) of rs2269714 elevated the expression levels of CD1A in blood samples [21]. The elevated expression of CD1A was associated with peripheral inflammation (skin inflammation, ulcerative colitis**,** and rheumatoid arthritis, etc.) [22–24]. Secreted by CD1A-reactive T cells, a variety of cytokines (IL-13, IL-22, IL-17A, TNF-α, IFN-γ and GM-CSF et al.) participated ininflammation, wound healing and defense against infection [24–26]. Inflammatory mediators or cytokines that are induced at the site of inflammation can enter the blood. These inflammatory signals could spread into central nervous system (CNS) through microglia [27]. Macrophages and microglia (cells of the mononuclear phagocyte lineage) play key roles in inflammation of chronic neurodegenerative disease [28]. It has been reported that peripheral immune stimuli can lead to differential epigenetic reprogramming of macrophages and microglia, cause long-term alterations in the brain immune response, and then affect the severity of miscellaneous neurodegenerative diseases, including AD [29]. Interestingly, this immune memory can also be elicited by individual cytokines [29]. In miscellaneous neurodegenerative diseases (AD, PD, and amyotrophic lateral sclerosis (ALS)), neuroinflammation is typified by a reactive morphology of glial cells (astrocytes and microglia) [30]. Moreover, the roles of peripheral inflammation in the development of multi-infarct dementia and AD have also been reported [31]. In summary, previous studies have indicated that the CD1A-related immune activation and peripheral inflammation may be important mechanisms contributing to neurodegenerative diseases.

As an important neuroimaging biomarker of metabolic abnormalities, [^18^F] FDG-PET can reflect the magnitude of cerebral hypometabolism [32]. Peripheral inflammation has been reported to reduce glucose metabolism in human medial temporal lobe (MTL) and impair human spatial memory [33]. But the molecular mechanism through which rs16840041 could affect human MTL function has not been studied yet. The decline rates of [^18^F] FDG were significantly associated with minor allele (A), further indicating the potential role of these SNPs in neurodegenerative diseases. Moreover, previous studies suggested that anti-CD1a antibody can reduce inflammation, indicating that blocking the interaction of CD1a with receptors on T cells could be a potential treatment for neurodegenerative diseases [34]. While the specific biological pathways underlying the role of *CD1A* in the vulnerability of neurodegenerative diseases require further investigation, the results reported here suggest that *CD1A* may be important for monitoring dementia progression at the individual level and evaluating early indicators of dementia. Moreover, those results also suggest that *CD1A* should be considered as a potential therapeutic target in dementia.

### Limitations

Several potential limitations of this report are as follows. First, the GWAS was conducted with modest samples sizes which restricted stratified analyses for each diagnostic group. Furthermore, we didn’t replicate these findings in an independent cohort due to limited data. Third, our sample was restricted to non-Hispanic white participants. We didn't explore the diversity among different populations.

## METHODS

### ADNI dataset

All participants were from the ADNI database which included three protocols (ADNI 1, ADNI 2 and ADNI Grand Opportunities (ADNI GO)). The ADNI database has recruited more than 1500 participants, including normal, MCI and AD subjects at present. ADNI was launched in 2003 by the National Institute on Aging, the National Institute of Biomedical Imaging and Bioengineering, the Food and Drug Administration, private pharmaceutical companies and nonprofit organizations. ADNI data (MRI and PET images, genetics, cognitive tests, and data on cerebrospinal fluid (CSF) and blood biomarkers) are disseminated by the Laboratory for Neuro Imaging at the University of Southern California. Informed consent was obtained from study participants, and the study was approved by the local institutional review board at each participating site. More information is available on the website of ADNI (http://adni.loni.usc.edu/).

### Participants

In this study, 545 subjects (healthy controls (HC) 224, MCI 321 at baseline) whose data met all QC criteria were included from the ADNI cohort. The demographic data and rate of change in plasma NFL in each group were summarized in Table 1.

The full cohort with GWS data and at least one follow-up for plasma NFL data included 614 participants. All the analyses were restricted to non-Hispanic white participants (n=559) to reduce the potential bias from population stratification. Population substructure and cryptic relatedness were checked with genomic identity-by-descent and multidimensional scaling (MDS) components and 5 participants were removed (Supplementary Figure 1). The QC of the rate of change in plasma NFL resulted in 545 valid samples. Moreover, ADNI samples showed tight clustering with individuals of European ancestry in MDS plot overlaid on HapMap samples (Supplementary Figure 2).

### Plasma measurements and QC

Plasma NFL was analyzed using the ultrasensitive Single Molecule array (Simoa) technique as previously described [35]. The assay used a combination of monoclonal antibodies and purified bovine NFL as a calibrator. Analytical sensitivity was < 1.0 pg/mL, and the NFL levels in all tested samples were above the detection limit. Changes in the NFL levels of the subjects were measured longitudinally. Further QC was performed to reduce the potential influence of extreme outliers on statistical results. Mean (-0.02 pg/ml·year) and standard deviations (SD) (1.97 pg/ml·year) of longitudinal rates of change in plasma NFL levels were calculated. Participants who had extreme outliers (<3-fold or >3-fold SD from the mean value) were removed from the analysis. This step removed 9 subjects.

### Genotyping and QC

The ADNI-1, ADNI-2, and ADNI-GO samples were genotyped with the Human 610-Quad BeadChip, Illumina Human Omni Express BeadChip and Ilumina Omni 2.5M BeadChip, respectively. PLINK software (version 1.07) was used in this step. The following criteria were utilized to perform a stringent QC assessment: call rates for individuals and SNPs were restricted to> 95%; minor allele frequencies (MAF) were restricted to > 0.05; p value for Hardy-Weinberg equilibrium test was restricted to > 0.001. An *APOE* genotyping kit was used to identify *APOE* alleles (polymorphisms rs7412 and rs429358) [36].

### Clinical disease progression

In the longitudinal study, HC and MCI participants were classified into either stable group or group of clinical disease progression (cognitive decline). Participants were designated as having clinical disease progression if their clinical classification or global CDR/MMSE score changed (HC subjects converted to MCI or AD, or their global CDR scores rose to 0.5 or more; MCI subjects lost more than 3 points between first and last MMSE administrations, converted to AD at follow-up, or got a score less than 24 on the last MMSE) [37–39]. If the above criteria have not been met at follow-up, participants were deemed stable.

### Post hoc analyses of other AD-related phenotypes

Genome-wide significant SNPs were further evaluated for associations with the rate of change in ADAS11, MMSE, [^18^F] FDG and the volume of AD-related brain regions (hippocampus and entorhinal cortex) using ADNI data. Designed to assess the severity of cognitive impairment, ADAS11 involves constructional and ideational praxis, language production and comprehension, learning and memory, and orientation [40]. The MMSE provides a global measure of mental status and involves language, recall, attention and calculation, orientation as well as registration [41]. Brain glucose metabolism, measured by [^18^F] FDG-PET, is associated with cognitive state [42]. [^18^F] FDG-PET scans were acquired and pre-processed using regions of interest (ROIs) (angular, temporal, and posterior cingulate) approach as described previously [43]. These ROIs were averaged together into a composite ROI which was used in [^18^F] FDG analyses. The volume of AD-related brain regions has been reported to be closely associated with cognitive state. The segmentation and analysis of cerebral images were performed using FreeSurfer version 5.1 (http://surfer.nmr.mgh.harvard.edu/). Longitudinal brain MRI scans and clinical data were downloaded from the ADNI public database (http://adni.loni.usc.edu/).

### Statistical analyses

Linear mixed models were utilized to compute longitudinal rates of change in the plasma NFL levels. These models were adjusted for age (*P*<0.001), diagnosis (*P*<0.001), and marital status (*P*<0.001). From these models, we estimated the mean rates of change for the whole samples. Using these longitudinal rates, we then fitted linear regression models using PLINK (version 1.07) [44]. An additive genetic model (i.e., dose-dependent effect of the minor allele) was utilized in those genetic association studies. As described above, the phenotype was the plasma NFL change rates extracted from the mixed effects models after adjustment for age, diagnosis, and marital status. The association analysis was additionally adjusted for the first two principal components (PCs) calculated by genome-wide complex trait analysis (GCTA) [45]. The thresholds of p< 1×10^−5^ and p< 5×10^−8^ were used for suggestive and genome-wide significant associations respectively [46]. Genome-wide associations were visualized with the R (version 3.5.1) package qqman [47]. Regional associations were visualized with the Locus Zoom web tool [48]. The association of mean annual changes in plasma NFL and A allele was tested using a linear regression model adjusting for age, diagnosis, and *APOE4* status.

Kaplan-Meier survival analysis of clinical disease progression was plotted based on rs16840041 genotypes. Log-rank test was used to compare the survival distributions of the different genotype subgroups. Cox proportional hazards models (adjusted for age, diagnosis, *APOE4* status) were used to test the predictive ability of the rs16840041genotypes for clinical disease progression. Linear mixed models were also used to estimate associations between the rs16840041 genotypes and the change rates of other AD-related phenotypes. All models were fitted with the lmer function in the R *lme4* package (version 1.1-18-1). Estimates and 95% CIs were based on parametric bootstrapping of the fitted models by use of the sim function in the *arm* package (version 1.10-1) with 10 000 replicates [49].

## CONCLUSIONS

In summary, we identified the associations of the three SNPs (rs16840041, rs2269714 and rs2269715) within *CD1A* with the increase in plasma NFL levels, faster decline of [^18^F] FDG and higher risk of cognitive decline among non-demented elders. These findings provide insights into the relationship of genetic variants with change rates of plasma NFL and AD-related phenotypes. The *CD1A* should be further investigated as a gene of interest in neurodegenerative diseases and as a potential target for monitoring disease trajectories and treating disease.

## Supplementary Material

Supplementary Figures

Supplementary Tables
