# Supplementary material for: Genome-wide association study identifies CD1A associated with rate of increase in plasma neurofilament light in non-demented elders
Source: Aging (Albany NY). 2019 Jul 11;11(13):4521–35. doi: 10.18632/aging.102066 (PMC6660034; doi:10.18632/aging.102066)
Supplement: Supplementary Figures [file aging-11-102066-s002.pdf]

SUPPLEMENTARY FIGURES

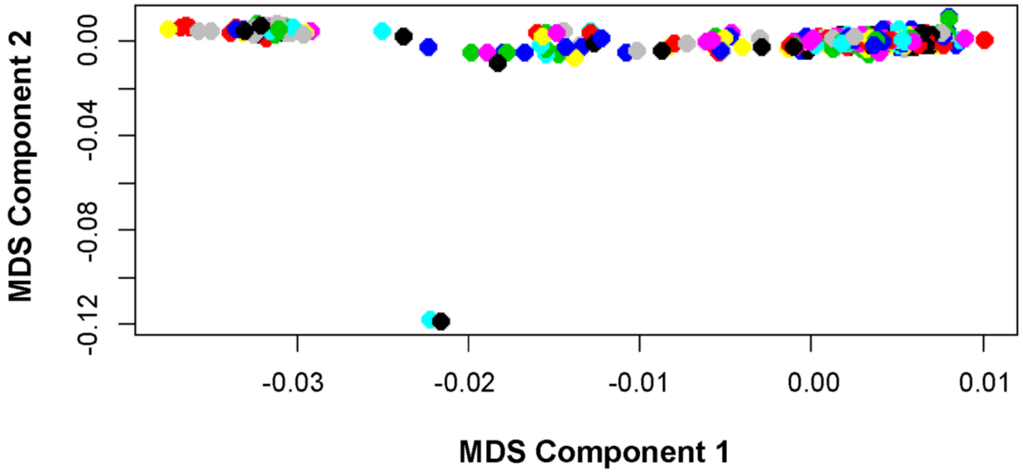

Supplementary Figure 1. MDS plot of ADNI non-Hispanic Caucasian samples. Samples seemed to form loose clusters and two samples were outliers based on the second MDS component (at bottom of plot; 031_S_4032 and 031_S_4203), suggesting potential population substructure. To check for cryptic relatedness, which can confound GWAS studies, pairwise identity-by-descent fraction (π) between each pair of samples were calculated using PLINK. Three related sample pairs were identified (137_S_4466 and 021_S_0159, π = 0.50; 023_S_0058 and 023_S_4035, π = 0.48; 024_S_2239 and 024_S_4084, π = 0.42), which are probably first-degree relatives. Optionally, we remove one member of each pair. No other cryptic relations were identified from the sample, at a threshold of $\pi > 0.05$.

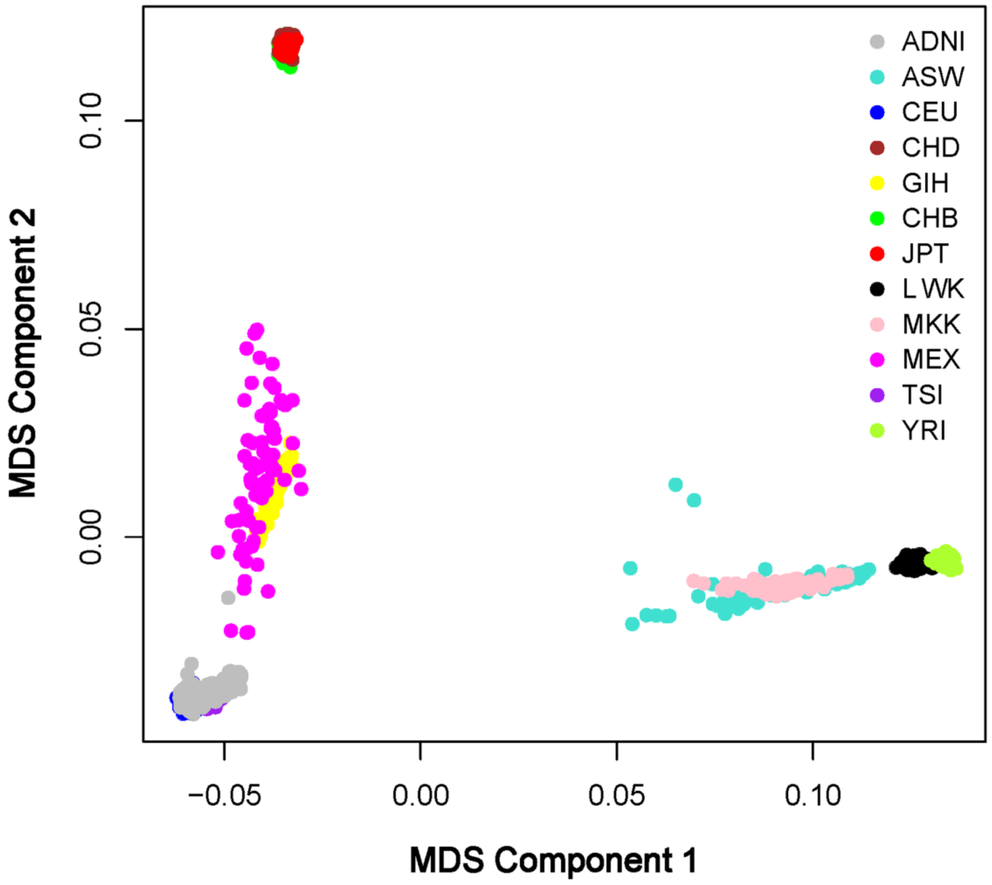

Supplementary Figure 2. MDS plot of ADNI samples overlaid on HapMap samples. The ancestry of the HapMap participants is shown by the point color. The outlying point represents Participant 116_S_1315 who is likely of mixed ancestry. Abbreviations: MDS, multidimensional scaling; ADNI, Alzheimer's Disease Neuroimaging Initiative; ASW, African ancestry in Southwest USA; CEU, Utah residents with Northern and Western European ancestry from the CEPH collection; CHB, Han Chinese individuals from Beijing, China; CHD, Chinese in Metropolitan Denver, Colorado; GIH, Gujarati Indians in Houston, Texas; JPT, Tokyo, Japan; LWK, Luhya in Webuye, Kenya; MEX, Mexican ancestry in Los Angeles, California; MKK, Maasai in Kinyawa, Kenya; TSI, Tuscans in Italy; YRI, Yoruba in Ibadan, Nigeria.

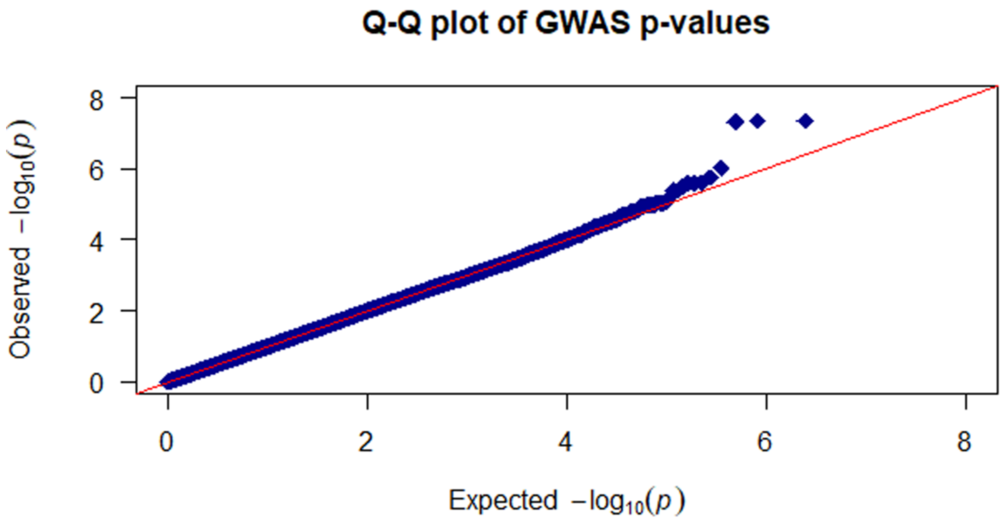

Supplementary Figure 3. The quantile-quantile (QQ) plot shows the negative logarithm of the observed and the expected p-value for each SNP.

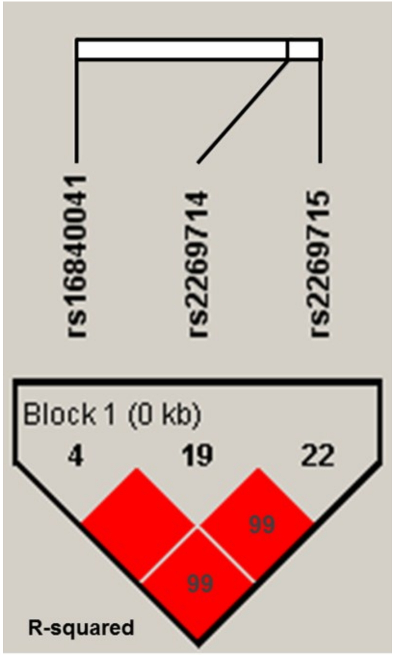

Supplementary Figure 4. Linkage-disequilibrium (LD) analysis of the variants rs16840041, rs2269714 and rs2269715 in *CD1A*. Pairwise linkage disequilibrium analysis shows r^2 (×100) values. The LD plots were generated using the Haploview software v4.2.
