# Supplementary material for: Genome-wide association study identifies CD1A associated with rate of increase in plasma neurofilament light in non-demented elders
Source: Aging (Albany NY). 2019 Jul 11;11(13):4521–35. doi: 10.18632/aging.102066 (PMC6660034; doi:10.18632/aging.102066)
Supplement: Supplementary Tables [file aging-11-102066-s001.pdf]

SUPPLEMENTARY TABLES

Supplementary Table 1. Suggestive SNPs in GWAS.

CHR	BP	SNP	MAF	Closest Gene	SNP Type/Location	*P* values
17	18055903	rs74361457	0.017	*MYO15A*	intron	9.52E-07
17	80461935	rs8078417	0.31	*NARF*	intergenic	1.75E-06
7	66903871	rs62465226	0.354	*LOC105375337*	intergenic	2.51E-06
7	66904395	rs6948216	0.393	*LOC105375337*	intergenic	2.51E-06
7	66908992	rs4618582	0.404	*LOC105375337*	intergenic	2.51E-06
7	66902955	rs7785167	0.413	*STAG3L4*	intergenic	3.16E-06
7	66898482	rs4357188	0.355	*STAG3L4*	intergenic	4.05E-06
7	66901317	rs12666354	0.358	*LOC105375337*	intergenic	4.05E-06
17	18227081	rs921986	0.323	*SMCR8*	intron	6.27E-06
17	18164404	rs2605142	0.2963	*MIEF2*	intron	8.30E-06
17	18228605	rs4925172	0.324	*SMCR8*	intron	9.11E-06
17	18231998	rs1979276	0.324	*SHMT1*	intron	9.11E-06
7	66896600	rs6460344	0.127	*LOC105375337*	intergenic	9.35E-06

Abbreviations: BP, base pair (variant position); CHR, chromosome; MAF, minor allele frequency; SNP, single nucleotide polymorphism

Supplementary Table 2. Baseline demographic characteristics and rs16840041 genotypes as predictors of time to clinical disease progression.

Characteristic	Hazard ratio (95% CI)	Wald χ^2 1	p value
genotype	1.63(1.12–2.36)	6.60	0.010
age	1.03(1.01–1.05)	6.66	0.010
diagnosis	1.45(1.24–1.70)	20.63	<0.001
APOE4 +	1.72(1.36–2.05)	24.04	<0.001

Cox proportional hazard models were used to assess the ability of demographic variables (age, diagnosis, and *APOE4* status) to predict clinical disease progression of AD over the 1-10 year follow-up period.
Abbreviations: CI, confidence intervals; *APOE*, apolipoprotein E.
